# Interaction of Water and Oxygen Molecules with Phosphorene: An Ab Initio Study

**DOI:** 10.3390/molecules28083570

**Published:** 2023-04-19

**Authors:** Francesca Benini, Nicolò Bassoli, Paolo Restuccia, Mauro Ferrario, Maria Clelia Righi

**Affiliations:** 1 Department of Physics and Astronomy, Alma Mater Studiorum—University of Bologna, 40127 Bologna, Italy; 2 Department of Physics, Informatics and Mathematics, University of Modena and Reggio Emilia, 41125 Modena, Italy

**Keywords:** water, phosphorene, adsorption, hydrophilicity

## Abstract

Phosphorene, the 2D form of black phosphorus, has recently attracted interest for optoelectronic and tribological applications. However, its promising properties are affected by the strong tendency of the layers to oxidize in ambient conditions. A significant effort has been made to identify the role of oxygen and water in the oxidation process. In this work, we introduce a first-principles study of the phosphorene phase diagram and provide a quantitative estimate of the interaction of pristine and fully oxidized phosphorene layers with oxygen and water molecules. Specifically, we study oxidized layers with oxygen coverages of 25% and 50% that keep the typical anisotropic structure of the layers. We found that hydroxilated and hydrogenated phosphorene layers are both energetically unfavorable, leading to structural distortions. We also studied the water physisorption on both pristine and oxidized layers, finding that the adsorption energy gain doubled on the oxidized layers, whereas dissociative chemisorption was always energetically unfavorable. At the same time, further oxidation (i.e., the dissociative chemisorption of O${}_{2}$) was always favorable, even on oxidized layers. Ab initio molecular dynamics simulations of water intercalated between sliding phosphorene layers showed that even under harsh tribological conditions water dissociation was not activated, thus further strengthening the results obtained from our static calculations. Overall, our results provide a quantitative description of the interaction of phosphorene with chemical species that are commonly found in ambient conditions at different concentrations. The phase diagram that we introduced confirms the tendency of phosphorene layers to fully oxidize due to the presence of O${}_{2}$, resulting in a material with improved hydrophilicity, a piece of information that is relevant for the application of phosphorene, e.g., as a solid lubricant. At the same time, the structural deformations found for the H- and OH- terminated layers undermine their electrical, mechanical, and tribological anisotropic properties and, therefore, the usage of phosphorene.

## 1. Introduction

Phosphorene, the single-layer form of black phosphorus, emerged in 2014 as a new member of the 2D class of materials [1]. Unlike graphene, phosphorene is a p-type semiconductor characterized by a direct band gap, which can be tuned by changing the number of layers [2] or by means of an in-plane strain [3]. Together with that, its high carrier mobility and anisotropic electrical and thermal conductivity paved the way for a variety of possible applications in optoelectronic devices [4]. As with other layered materials, such as graphene and MoS${}_{2}$ [5,6,7], phosphorene is also appealing for tribological applications. Its mechanical and lubricating properties were shown to be promising in reducing nano-asperity adhesion [8,9,10,11,12]. Moreover, the peculiar puckered structure of phosphorene gives rise to unique anisotropic tribological features [13,14], and superlubricity at the nanoscale was predicted due to the dramatic reduction in the interlayer shear strength obtained with a perpendicular orientation of the layers [15,16].

Phosphorene usage in the above-described fields is, however, jeopardized by its strong tendency to degrade under ambient conditions [17,18,19,20,21]. The atomic-scale mechanisms of oxidation of phosphorene have been investigated theoretically [22,23,24,25,26] and experimentally [18,27,28], giving rise to different interpretations of the role played by water. Ultimately, oxidation was found to be induced by the interaction with O${}_{2}$, whereas water alone does not seem to react with pristine phosphorene, even if it may act as a catalyst for O${}_{2}$-induced oxidation [18,22]. While the interaction of pristine phosphorene with water has been extensively explored [29], the study of the hydrophilicity of oxidized phosphorene is limited to the effects of localized oxygen atoms chemisorbed on the surface [18,22,26,30], although an understanding of the hydrophilic behavior of fully oxidized layers would be important for most applications. In optoelectronics, protection mechanisms against degradation, such as encapsulation [31], oxygen plasma etching [32], solvent passivation [33], and pulsed laser exfoliation [34], have been explored. In tribology, the interaction of layered materials with molecules present in the environment can deeply affect their lubricating capabilities. Humidity, for example, improves the tribological performance of graphite, while it is detrimental for MoS${}_{2}$ lubricity [35,36,37,38]. Experiments on black phosphorus showed that the layer oxidation improved its friction reduction performance in aqueous environments [9,10,11,39]. Moreover, density functional theory calculations showed that the anisotropy of phosphorene mechanical properties could be tuned via oxidation [40].

In typical working environments, stable configurations for surfaces result from adsorption and desorption of chemical species from the surrounding atmosphere. Therefore, a combination between statistical thermodynamics and ab initio calculations is necessary to properly describe these processes. In this context, ab initio thermodynamics techniques proved to be powerful tools for theoretically determining the stability of surfaces under the effects of environmental gases at non-zero temperatures and pressures [41,42,43]. This type of analysis has been exploited in a variety of fields in which the stability of materials in realistic working conditions is crucial and needs to be considered, such as in the study of corrosion mechanisms [44], catalysis [45,46,47], and tribology [48,49] in realistic environments.

To identify the most likely termination of phosphorene in the presence of oxygen and water molecules in different concentrations, we derived its phase diagram by means of ab initio thermodynamics. Finally, we studied the adsorption and dissociation of water and oxygen molecules on pristine and homogeneously oxidized layers to evaluate the effect of oxidation on the layers hydrophilicity. Moreover, we performed ab initio molecular dynamics simulations of water molecules confined between pristine phosphorene interfaces to explore the effects of harsh tribological conditions on phosphorene when interacting with water.

## 2. Results and Discussion

### 2.1. Relative Stability of Phosphorene Layers with Different Terminations

**Figure 1:**
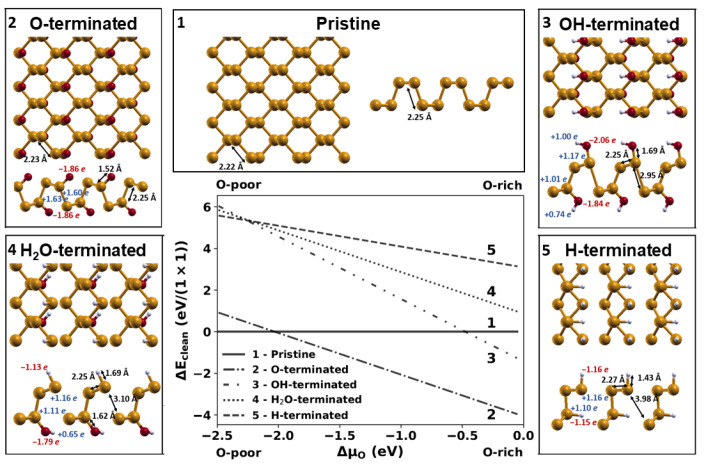
Phase diagram of phosphorene with different terminations at 50% coverage. In the surrounding panels representing the optimized structures, the most relevant bond lengths and partial charges are indicated. Yellow, red, and light-gray colors are used for phosphorous, oxygen, and hydrogen atoms, respectively.

As a first step in our study, we analyzed the structure and stability of phosphorene layers with different terminations by constructing a phase diagram. The considered structures are shown in Figure 1; they include pristine phosphorene, O-, H-, and OH-terminated layers, and a combined termination obtained by splitting water molecules into -H and -OH fragments. In all of the cases, we considered a 50% coverage of surface P atoms. For this oxidized system, which is denoted as PO${}_{0.5}$ from now on and is shown in panel 2 in Figure 1, the O atoms bind to the P atoms through a lone pair, and the chemical groups are equally distributed above and below the P layers, as previously suggested for the most stable binding configuration for oxygen on phosphorene [18,23,40,50,51]. Bond lengths and partial charges are indicated for all of the optimized structures shown in Figure 1. In particular, a negative value of the partial charge (in blue in Figure 1) describes electron accumulation over the considered atom, whereas a positive value (in red in Figure 1) indicates a depletion. Partial charge values are expressed in units of elementary charge, and they can be interpreted as a description of the charge transfer due to electronegativity differences among atoms connected by chemical bonds.

The phase diagram, whose construction is explained in Section 3 shows that the O-termination is more stable than all other possible terminations, and it is, in general, more stable than the pristine layer, except in conditions that are extremely poor in terms of oxygen. This result agrees with the experimental observation that phosphorene oxidation most easily occurs in air [18,27]. Interestingly, phosphorene oxidation preserves the peculiar geometry of 2D phosphorene, which can give rise to incommensurate low-friction layer orientations [16]. The P-O bonds are shorter than the P-P bonds and polar due to the difference in the electronegativity of the species involved, respectively, of 2.19 for P and 3.44 for O. The other considered molecular groups (H-, OH-, and H${}_{2}$O-terminations) brought a significant distortion to the phosphorene lattice structure, with P-P bonds being up to 80% longer than those of pristine phosphorene, which are, therefore, easier to break. The adsorption of hydrogen or water fragments can lead to P-P bond breaking in the phosphorene structure, which will then be split into two separate parts. The characteristic electronic, mechanical, and tribological properties of phosphorene are deeply affected by this structural change, with detrimental effects on the applications.

To complete the stability analysis of terminated phosphorene, we considered whether passivating only one side of phosphorene could affect the previous picture.

**Figure 2:**
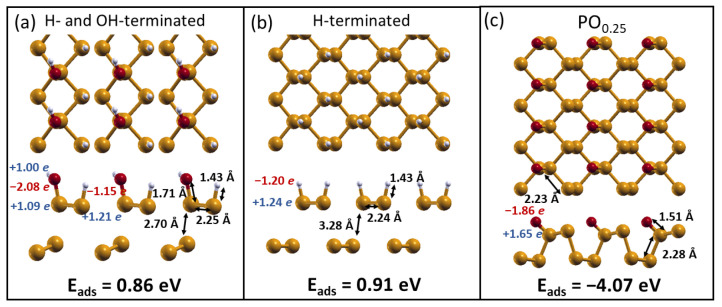
Top (**upper panel**) and lateral (**lower panel**) views of the optimized structures of one-sided functionalized phosphorene substrates covered in -OH and H (**a**), H (**b**), and O (**c**) atoms. The adsorption energies for each molecule are reported.

As shown in Figure 2, we found similar results to those shown before, where 25% oxidized phosphorene (denoted as PO${}_{0.25}$ from now on) is the most stable system with lower distortions of the phosphorene lattice structure. For the H- and OH-/H-terminations, however, strong structural deformations were found; therefore, they were discarded from the study of the interaction with water.

### 2.2. H_2_O Interacting with Oxidized Phosphorene

**Figure 3:**
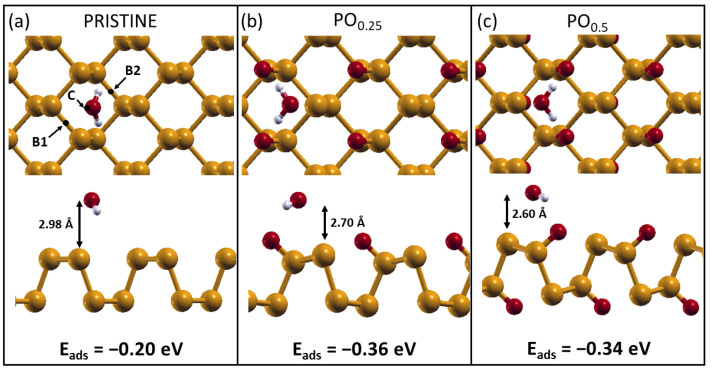
Top (**upper panel**) and lateral (**lower panel**) view of the optimal configurations for water adsorption on the three phosphorene substrates: pristine phosphorene (**a**) and oxidized phosphorene with an oxygen coverage of, respectively, 25% (**b**) and 50% (**c**). The three adsorption sites tested are represented as well: short bridge (B1), center (C), and long bridge (B2). The relevant lengths are reported, together with the three adsorption energies.

After identifying that oxidized phosphorene is the most stable phase of the layer for a wide range of conditions, we studied how a complete oxidation can affect phosphorene hydrophilicity by considering water physisorption and dissociative chemisorption on oxidized layers. To identify the most stable configuration for molecular adsorption, we positioned the molecule in three different symmetry sites on the surface, considering four different molecular orientations for each site. The results obtained for pristine phosphorene, PO${}_{0.25}$, and PO${}_{0.5}$ are shown in Figure 3.

The optimal configurations obtained for water adsorption on the three different substrates are reported in Figure 3. As can be seen, the energy gain associated with the adsorption of a single water molecule almost doubles when passing from pristine to oxidized layers, reaching values of 0.36 eV for the PO${}_{0.25}$ system and 0.34 eV for the PO${}_{0.5}$. As is visible in the lateral views of Figure 3, a shorter distance from the surface is generally associated with a stronger interaction. The adsorption energy obtained for pristine phosphorene (0.20 eV) is in reasonable agreement with previous calculations, where slightly different computational parameters were employed [18,29]. Overall, our results show that a fully oxidized phosphorene layer promotes the attraction of water molecules through the formation of H-bonds, thus increasing the layer hydrophilicity. This is a confirmation of previous calculations performed on phosphorene with localized oxidation [29], but it is more realistic and general thanks to the complete oxidation present in our systems, as the structural modifications and surface polarization induced by O-adsorption are fully taken into account. While for optoelectronic devices, this enhanced hydrophilicity might be harmful, in tribological conditions, the formation of a water layer on oxidized black phosphorus was experimentally proven to be beneficial [9]. This mechanism is at play also in the case of silicon-doped diamond-like carbon (DLC) [52].

### 2.3. H_2_O and O_2_ Dissociation

Once it was proven that layer oxidation promotes water attraction, we investigated whether it may also enable water dissociation. To this end, we identified the most stable dissociative chemisorption configurations on the three considered layers and calculated the energy cost/gain, $\Delta E_{\left(chem-phys\right)}$, for passing from the initial physisorption state to the dissociated one.

**Figure 4:**
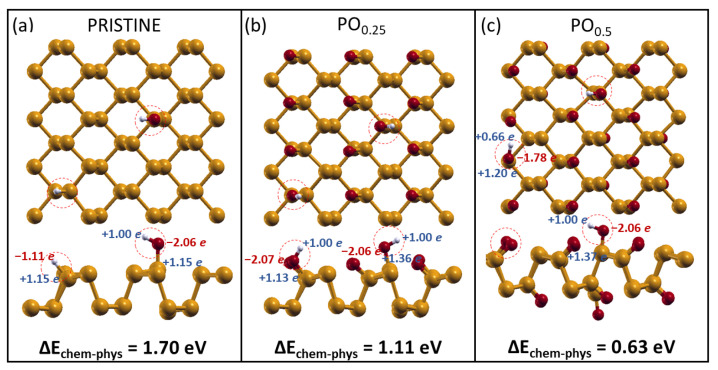
Water dissociation on the three phosphorene substrates: (**a**) pristine phosphorene and oxidized phosphorene with an oxygen coverage of, respectively, (**b**) 25% and (**c**) 50%. Above, the top view is shown, while below, the lateral views are reported. The partial charges transferred among atoms are reported alongside the three adsorption energies.

The configurations obtained for H${}_{2}$O dissociation on the three phosphorene substrates are reported in Figure 4. While for pristine phosphorene, the configuration is that already seen in Section 2.1, where the -OH and -H fragments are chemisorbed on dangling P, in the case of oxidized phosphorene, the species are chemisorbed on P and O atoms, respectively, giving rise to the presence of two -OH groups per cell. Our results show that water dissociation is not favorable on phosphorene, resulting in an endothermic process for all three cases, even though higher degrees of oxidation seem to reduce the energy required to dissociate water molecules.

**Figure 5:**
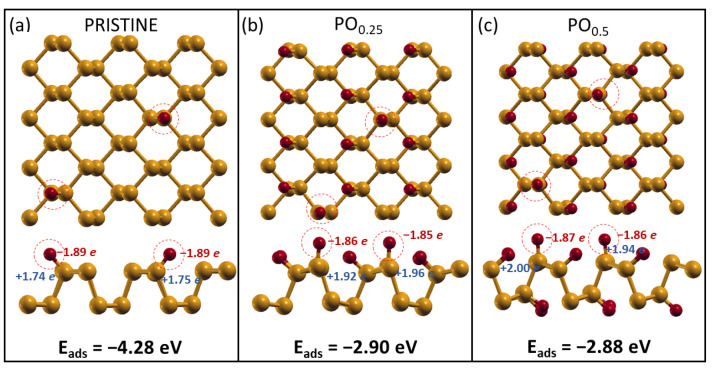
Oxygen dissociation on the three phosphorene substrates: (**a**) pristine phosphorene and oxidized phosphorene with an oxygen coverage of, respectively, (**b**) 25% and (**c**) 50%. Above, the top view is shown, while below, the lateral views are reported. Partial charges on atoms are displayed, together with the three adsorption energies.

We performed the same analysis for O${}_{2}$ dissociation to see if it differed from the case of water. The configurations obtained for these systems and the associated energy gains are reported in Figure 5. The O${}_{2}$ dissociative adsorption increases the oxygen coverage, and up to this degree of oxidation, the lattice deformation still remains small. We found that further O${}_{2}$ dissociation on oxidized layers is still energetically favorable. However, the energy gain decreases because of the electrostatic repulsion among O atoms on the surface. This result is consistent with the literature [51], and it might be relevant for tribological applications, where the usage of degraded phosphorene as a lubricant additive has already been proven to be successful in reducing friction, leading to superlubricity in water-based lubrication [9,10,11,39].

### 2.4. H_2_O Intercalation within Phosphorene Layers

In analogy with previous studies performed for graphene and MoS${}_{2}$ [38], we evaluated the stability of water intercalation within pristine phosphorene layers. As a first step, we considered a single water molecule and compared its adsorption energies above and within a phosphorene bilayer. The molecule was positioned in the most stable adsorption site, which was identified before, and a supercell with a 4 × 3 in-plane size was employed for these calculations, as well as in the molecular dynamics simulations described in the following section.

**Figure 6:**
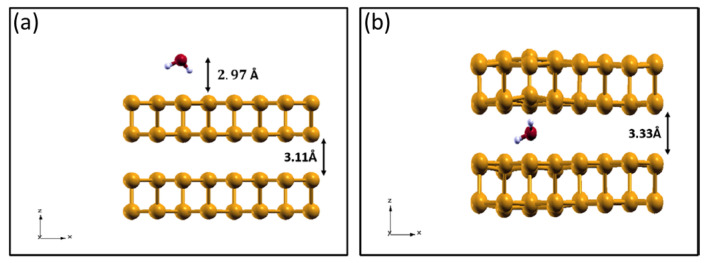
Side view of water adsorbed (Panel (**a**)) above and (Panel (**b**)) intercalated within the phosphorene bilayer.

As can be seen from the optimized configurations shown in Figure 6, molecule intercalation increases the interlayer spacing, particularly in the region around the molecule, where the interlayer distance reaches 3.93 Å, causing the out-of-plane deformation of the layers, as is visible in Figure 6b. This configuration is energetically unfavorable, as the adsorption energy per unit area is equal to 0.028 J/m${}^{2}$. On the contrary, the molecular adsorption over the phosphorene bilayer produces an energy gain of −0.041 J/m${}^{2}$ (the adsorption energy is calculated according to Equation (2), considering the phosphorene bilayer as a substrate).

The instability of the intercalated configuration can be reduced by increasing the water coverage. In this way, it is possible to compensate for the reduced layer–layer interaction by increasing the molecule–layer and molecule–molecule interactions. To this end, we considered a 25% and a 50% coverage of interfacial molecules (the coverage is calculated as the ratio of the occupied and available adsorption sites).

**Figure 7:**
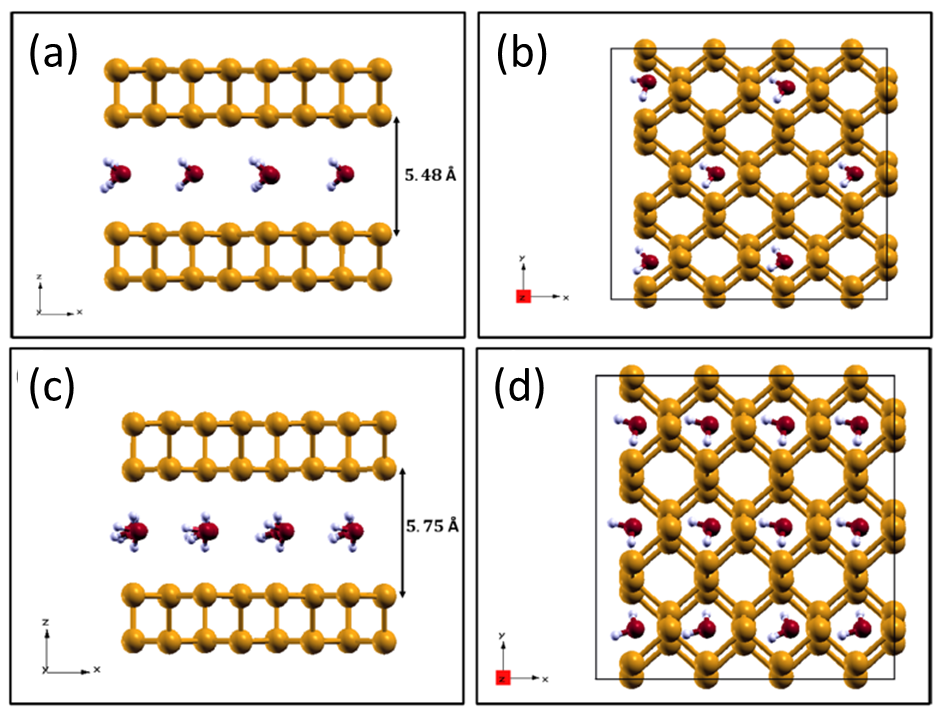
Side view (Panels (**a**,**c**)) and top view (Panels (**b**,**d**)) of a phosphorene bilayer with 25% (upper panels) and 50% (lower panels) of superficial intercalated water coverage.

As can be seen from the optimized configurations of the two systems reported in Figure 7, the phosphorene layers did not undergo any appreciable deformation, and the interlayer spacing stayed uniform. Water intercalation becomes favorable with associated energy gains of −0.34 J/m${}^{2}$ and −0.56 J/m${}^{2}$, respectively. The extra binding contribution gained by the water molecules is mainly due to their mutual interaction through hydrogen bonds.

### 2.5. Intercalated Water in Harsh Tribological Conditions

The results described in Section 2.3 suggest that water dissociation due to the interaction with the phosphorene layers is an endothermic process. However, it is well known that the harsh conditions that are typical of tribological interfaces, which include molecular confinement, applied mechanical stresses, and frictional heating, may sometimes favor and speed up the activation of chemical processes that are otherwise difficult to observe on an open surface in static conditions. To verify whether these conditions can favor dissociative adsorption of water on phosphorene, we performed ab initio molecular dynamics simulations (AIMDs) of sliding phosphorene layers with intercalated water molecules at ambient temperature and an ultra-high pressure of 10 GPa. After a preliminary structural optimization of the system under load, the two layers were slid in opposite directions.

**Figure 8:**
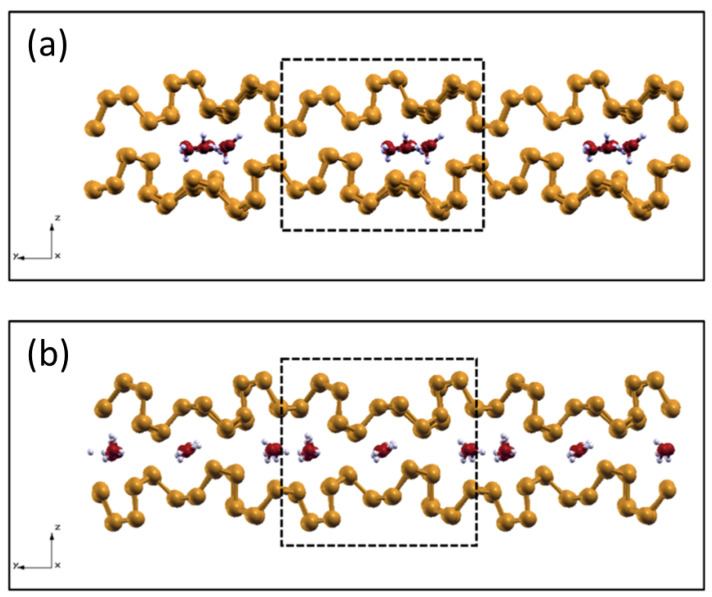
Side view of the phosphorene interfaces covered by 25% (**a**) and 50% (**b**) of water molecules during 8.7 ps and 11.6 ps of sliding dynamics under extreme pressure conditions.

As can be seen in Figure 8a (Figure 8b) where the configuration assumed by the interface covered by 25% (50%) during sliding is presented, we did not observe any tribologically induced dissociation of the water molecules. On the contrary, during the dynamics simulations, the water molecules tended to cluster in order to form more hydrogen bonds. This phenomenon was particularly evident in the 25% coverage system, where the molecules gathered on one side of the supercell (marked with a dashed line Figure 8a), leaving the rest of the interface uncovered. In this region that was depleted of water molecules, the interplay of the high applied load and of the layer deformation induced by the intercalated molecules promoted the formation of P-P chemical bonds across the interface, which caused the phosphorene to rupture.

A different evolution was observed for the 50% coverage system, where the increased water coverage implied a much more uniform distribution of the molecules at the interface, which prevented direct contact between the phosphorene layers. Water molecules still tended to coalesce in clusters by means of their mutual attraction, but the cluster density was high enough to effectively behave as an (incomplete) water monolayer that separated the phosphorene layers and acted as a buffer with respect to the external applied normal load, as can be seen in Figure 8a,b. This enhanced the stability of the sliding interface.

A similar study was carried out by Levita et al. [53] by considering an interface composed of two sliding MoS${}_{2}$ layers subject to a normal load of a similar magnitude to that applied in this study, and it was intercalated with water molecules at 25% and a 50% coverages. They observed—analogously to what was observed for phosphorene—the formation of water clusters promoted by hydrogen bonding. In both cases, the intercalated molecules interlocked the two layers, hindering their relative motion. A similar effect was observed for the phosphorene bilayer with intercalated water at 25% coverage, where the molecules gathered to produce enhanced layer deformations that could end up in layer rupture. The case of 50% water coverage seemed, instead, to behave differently, showing enhanced structural stability and promoting relative sliding.

## 3. Materials and Methods

All of the calculations were performed by using spin-polarized density functional theory calculations, as implemented in version 6.8 of the Quantum ESPRESSO suite [54,55,56]. The generalized gradient approximation (GGA) within the Perdew–Burke–Ernzerhof (PBE) parametrization was exploited to describe the exchange-correlation functional [57]. To properly describe van der Waals interactions, the Grimme D2 dispersion correction scheme was used with the $s_{6}$ parameter, as suggested in the original paper ($s_{6}$ = 0.75) [58] and as already tested for phosphorene in previous works of our group [12,16]. The electronic wave function was expanded on a plane–wave basis with a cutoff of 50 Ry, whereas a cutoff of 400 Ry was chosen for the charge density. Ultra-soft pseudopotentials were employed for the description of the ionic species. Structural optimizations were carried out by using default criteria for energy and force convergence ($10^{-4}$ Ry and $10^{-3}$ Ry/Bohr, respectively), and a Gaussian smearing of 0.005 Ry was used to favor the optimization procedure, especially for the interaction between oxygen and phosphorous atoms. The preliminary optimization was performed on a unit cell of pristine phosphorene with a 12 × 9 × 1 Monkhorst–Pack k-point grid [59]. To study the interaction with water, a 4 × 3 orthorhombic supercell was used, where a 10 Å vacuum along the z direction was set in order to avoid undesired interactions among replicas. For the latter systems, we employed a 3 × 3 × 1 Monkhorst–Pack grid for the k-point sampling.

Ab initio molecular dynamics simulations in the Born–Oppenheimer approximation were performed within the same computational setup, except for the k-point sampling, which was only reduced to Γ. We used the MD module contained in the Quantum ESPRESSO package, which was modified to allow the imposition of a constant sliding velocity for a specific group of atoms in the systems. To achieve stationary conditions, the temperature was controlled with a set of thermostats acting on other groups of atoms while taking into account only the *thermal* part of the velocities, i.e., removing the group sliding velocity from the thermal kinetic energy calculation when needed [60]. The integration time step for the molecular dynamics simulations was set equal to dt = 30 a.u., i.e., dt ≈ 1.45 fs. The super cell contained a bilayer of phosphorene with periodic boundary conditions, where an additional amount of vacuum with a height equal to 10 Å was included along the vertical direction to separate the replicas. Simulations were carried out under the effect of a normal load of 10 GPa and with a fixed sliding velocity for each phosphorene layer equal to ±100 m/s such that there was a relative layer velocity of 200 m/s at the interface. The initial velocities for each atom were sampled from a Maxwellian distribution corresponding to a temperature of 300 K, and the system was initially thermalized under a load by integrating the dynamics without any applied sliding velocity. After that, the evolution of the system under tribological conditions was carried on for 8000 steps, corresponding to a simulation time interval of approximately 11.6 ps.

The phase diagram was constructed by calculating the energy cost/gain, $\Delta E_{clean}$, to terminate the pristine phosphorene layers with O- and H-containing groups as follows:

(1) $$\Delta E_{clean}=\Delta E_{tot}-n_{H}\mu_{H}-n_{O}\mu_{O}$$

where $\Delta E_{tot}$ is the energy difference between the terminated and pristine phosphorene, $n_{H}$ ($n_{O}$) is the number of hydrogen (oxygen) atoms adsorbed over the layer, and $\mu_{H}$ ($\mu_{O}$) the chemical potential of hydrogen (oxygen). Considering the equilibrium conditions $\mu_{H_{2}O}=2\mu_{H}+\mu_{O}$, where $\mu_{H_{2}O}$ is the energy of a water molecule [48,49], the above relation can be written as a function of the chemical potential of the oxygen atom only. Its variation, $\mu_{O}=\frac{1}{2}\mu_{O_{2}}+\Delta \mu_{O}$, reflects the environmental conditions, which can change from O-rich ($\Delta \mu_{O}$ = 0) to O-poor ($\Delta \mu_{O}=\Delta E_{H_{2}O}$), where $\Delta E_{H_{2}O}$ is the formation energy of a $H_{2}O$ molecule calculated as $\Delta E_{H_{2}O}=\mu_{H_{2}O}-\frac{1}{2}\mu_{O_{2}}-\frac{1}{2}\mu_{H_{2}}$.

In the systematic study of the adsorption of water on phosphorene layers, three sites on the surface were tested for each substrate: center, short bridge (B1), and long bridge (B2), as shown in Figure 3. For each adsorption site, four different orientations for the water molecule were tested: “one leg down”, “two legs down”, oxygen down, and planar, where with “leg down”, one or more H atoms are meant to point directly at the surface. To model the isolated water and oxygen molecules, the same computational setup was used. The adsorption energy was calculated as:

(2) $$E_{ads}\left(n\right)=\frac{1}{n}\left[E_{tot}\left(n\right)-\left(E_{sub}+nE_{mol}\right)\right]\;,$$

where $E_{tot}\left(n\right)$ represents the total energy of the interacting systems composed of a substrate and *n* molecules, while $E_{sub}$ and $E_{mol}$ are the energies of the phosphorene substrate and the isolated molecules, respectively. In the study of oxidized substrates, this value can be seen as the energy associated with the dissociation of *n* O${}_{2}$ molecules on a phosphorene layer and can be compared to twice the values reported in [51], where the formation energies are expressed per single O atom on the surface. The reaction energy for dissociation was calculated as

(3) $$\Delta E_{\left(chem-phys\right)}=E_{chem}-E_{phys}\;,$$

where $E_{chem}$ is the energy associated with the fragments chemisorbed on the phosphorene layer, while $E_{phys}$ represents the energy calculated for the most favorable physisorption configuration. Negative (positive) values of $\Delta E_{\left(chem-phys\right)}$ indicate that the dissociative chemisorption is an exothermic (endothermic) process.

Finally, all of the considerations concerning charge transfer among substrates and chemisorbed species were realized by means of Bader charge analysis methods [61,62,63,64].

## 4. Conclusions

In this study, we identified the relative stability of different terminations of phosphorene by calculating its phase diagram from the first principles. Our results showed that oxidized phosphorene is the most stable phase for a wide range of chemical potentials, which is in agreement with the observed tendency of phosphorene to oxidize in the presence of oxygen molecules. The formation of phosphorene layers covered with -OH and -H fragments, resulting from the dissociation of H${}_{2}$O and H${}_{2}$ molecules, is energetically unfavorable and, therefore, unlikely to occur. We are not aware of any previous work presenting a phase diagram of phosphorene. The phase diagram describes the stability of different phosphorene terminations in a wide range of conditions that include the abundance of precursors, the temperature, and the pressure (which are taken into account according to the variation in the chemical potentials of the adsorbed species). The performed analysis showed that oxidized phosphorene is the most stable phase for this layered material in all of the considered conditions, apart from a situation in which oxygen is almost absent. Other terminations, such as hydrogenation and the termination resulting from the dissociative adsorption of water molecules, are not energetically favored; thus, they are not expected to form spontaneously. We believe that this information, which, to our knowledge, is not present in the literature, can be highly relevant for designing applications of phosphorene in different environments. By studying the effects of the interaction of water with the fully oxidized phosphorene layer, we found out that the energy associated with water adsorption almost doubles on the oxidized layers with respect to the pristine one, proving that oxidation enhances the hydrophilicity of this material.

We also found out that while the dissociation of a single water molecule is still unfavorable on oxidized substrates, further O${}_{2}$ dissociation is promoted without remarkable lattice distortions. The phosphorus oxides maintain the peculiar puckered phosphorene structure, which is at the basis of many of its anisotropic properties. Concerning O${}_{2}$ dissociation on oxidized layers, however, we obtained energy gains that were significantly lower than that obtained for pristine phosphorene (2.90–2.88 eV and 4.28 eV, respectively) due to the increasing electrostatic repulsion among O atoms on the surface.

Finally, we studied the intercalation of water molecules within phosphorene layers and found that the stability of the intercalated structures increases with the interfacial coverage. These structures were also simulated under the effects of harsh tribological conditions. Our ab initio MD simulations of sliding layers revealed that despite the extreme applied load, which is known to enhance chemical reactivity [53,60,65], water molecules do not dissociate when interacting with phosphorene. Confined water only tends to cluster because of hydrogen bonding, indicating that molecule–molecule interactions prevail over molecule–substrate interactions. These results are in agreement with the experiments reported in the literature. The protocol reported by Ren et al. [39] for producing partially oxidized black phosphorus nanosheets consists of dispersing bulk black phosphorus powder in a solution of water and hydrogen peroxide as a strong oxidizing agent, which is stirred for 30 min and then sonicated for 2 h. This procedure indicates that pure water alone cannot be used to chemically degrade black phosphorus.

Overall, our results provide a piece of information that might be useful for several different applications of phosphorene and, in particular, for the possible usage of oxidized phosphorene layers in tribology, a field in which the hydrophilicity of 2D materials has already been proven to be a major player that affects their tribological performance in a unique way specific to each kind of material.

## Data Availability

The data-sets generated and/or analyzed during the current study will be available in the Tribchem website, at the link (http://tribchem.it/?page_id=1663, accessed on 27 February 2023).

## References

[1] Churchill H.O., Jarillo-Herrero P. (2014). Phosphorus joins the family. Nat. Nanotechnol..

[2] Low T., Rodin A., Carvalho A., Jiang Y., Wang H., Xia F., Neto A.C. (2014). Tunable optical properties of multilayer black phosphorus thin films. Phys. Rev. B.

[3] Rodin A., Carvalho A., Neto A.C. (2014). Strain-induced gap modification in black phosphorus. Phys. Rev. Lett..

[4] Liu H., Du Y., Deng Y., Ye P.D. (2015). Semiconducting black phosphorus: Synthesis, transport properties and electronic applications. Chem. Soc. Rev..

[5] Elinski M.B., Liu Z., Spear J.C., Batteas J.D. (2017). 2D or not 2D? The impact of nanoscale roughness and substrate interactions on the tribological properties of graphene and MoS_2_. J. Phys. D Appl. Phys..

[6] Spear J.C., Ewers B.W., Batteas J.D. (2015). 2D-nanomaterials for controlling friction and wear at interfaces. Nano Today.

[7] Rosenkranz A., Liu Y., Yang L., Chen L. (2020). 2D nano-materials beyond graphene: From synthesis to tribological studies. Appl. Nanosci..

[8] Cui Z., Xie G., He F., Wang W., Guo D., Wang W. (2017). Atomic-scale friction of black phosphorus: Effect of thickness and anisotropic behavior. Adv. Mater. Interfaces.

[9] Galluzzi M., Zhang Y., Yu X.F. (2020). Mechanical properties and applications of 2D black phosphorus. J. Appl. Phys..

[10] Wu S., He F., Xie G., Bian Z., Luo J., Wen S. (2018). Black phosphorus: Degradation favors lubrication. Nano Lett..

[11] Wu S., He F., Xie G., Bian Z., Ren Y., Liu X., Yang H., Guo D., Zhang L., Wen S. (2020). Super-slippery degraded black phosphorus/silicon dioxide interface. ACS Appl. Mater. Interfaces.

[12] Losi G., Cutini M., Restuccia P., Righi M.C. (2022). Modeling phosphorene and MoS_2_ interacting with iron: Lubricating effects compared to graphene. J. Nanostruct. Chem..

[13] Tao J., Shen W., Wu S., Liu L., Feng Z., Wang C., Hu C., Yao P., Zhang H., Pang W. (2015). Mechanical and electrical anisotropy of few-layer black phosphorus. ACS Nano.

[14] Gong H., Zhu P., Si L., Zhang X., Xie G. (2018). “M-shape” nanoscale friction anisotropy of phosphorene. Comput. Mater. Sci..

[15] Wang W., Xie G., Luo J. (2018). Superlubricity of black phosphorus as lubricant additive. ACS Appl. Mater. Interfaces.

[16] Losi G., Restuccia P., Righi M. (2020). Superlubricity in phosphorene identified by means of ab initio calculations. 2D Mater..

[17] Kuriakose S., Ahmed T., Balendhran S., Bansal V., Sriram S., Bhaskaran M., Walia S. (2018). Black phosphorus: Ambient degradation and strategies for protection. 2D Mater..

[18] Huang Y., Qiao J., He K., Bliznakov S., Sutter E., Chen X., Luo D., Meng F., Su D., Decker J. (2016). Interaction of black phosphorus with oxygen and water. Chem. Mater..

[19] Island J.O., Steele G.A., van der Zant H.S., Castellanos-Gomez A. (2015). Environmental instability of few-layer black phosphorus. 2D Mater..

[20] Utt K.L., Rivero P., Mehboudi M., Harriss E.O., Borunda M.F., Pacheco SanJuan A.A., Barraza-Lopez S. (2015). Intrinsic defects, fluctuations of the local shape, and the photo-oxidation of black phosphorus. ACS Cent. Sci..

[21] Hyun C., Kim J.H., Lee J.Y., Lee G.H., Kim K.S. (2020). Atomic scale study of black phosphorus degradation. RSC Adv..

[22] Kumar J., Shrivastava M. (2022). First-principles molecular dynamics insight into the atomic level degradation pathway of phosphorene. ACS Omega.

[23] Ziletti A., Carvalho A., Campbell D.K., Coker D.F., Castro Neto A.H. (2015). Oxygen Defects in Phosphorene. Phys. Rev. Lett..

[24] Wang G., Slough W.J., Pandey R., Karna S.P. (2016). Degradation of phosphorene in air: Understanding at atomic level. 2D Mater..

[25] Kistanov A.A., Cai Y., Zhou K., Dmitriev S.V., Zhang Y.W. (2016). The role of H_2_O and O_2_ molecules and phosphorus vacancies in the structure instability of phosphorene. 2D Mater..

[26] Eslamibidgoli M.J., Eikerling M.H. (2018). Mechanical and chemical stability of monolayer black phosphorous studied by density functional theory simulations. J. Phys. Chem. C.

[27] St Laurent B., Dey D., Yu L., Hollen S. (2021). Atomic-Scale Investigation of Oxidation at the Black Phosphorus Surface. ACS Appl. Electron. Mater..

[28] Zhang T., Wan Y., Xie H., Mu Y., Du P., Wang D., Wu X., Ji H., Wan L. (2018). Degradation chemistry and stabilization of exfoliated few-layer black phosphorus in water. J. Am. Chem. Soc..

[29] Xu Y., Guo W. (2018). Optimal water adsorption on phosphorene. J. Alloy. Compd..

[30] Foroutan M., Bavani B.M., Boudaghi A. (2022). Controlled hydrophilization of black phosphorene: A reactive molecular dynamics simulation approach. Phys. Chem. Chem. Phys..

[31] van Druenen M. (2020). Degradation of Black Phosphorus and Strategies to Enhance Its Ambient Lifetime. Adv. Mater. Interfaces.

[32] Pei J., Gai X., Yang J., Wang X., Yu Z., Choi D.Y., Luther-Davies B., Lu Y. (2016). Producing air-stable monolayers of phosphorene and their defect engineering. Nat. Commun..

[33] Hanlon D., Backes C., Doherty E., Cucinotta C.S., Berner N.C., Boland C., Lee K., Harvey A., Lynch P., Gholamvand Z. (2015). Liquid exfoliation of solvent-stabilized few-layer black phosphorus for applications beyond electronics. Nat. Commun..

[34] Zhang J., Shin H., Lu W. (2019). Highly ambient-stable few-layer black phosphorene by pulsed laser exfoliation and HEMM. Chem. Commun..

[35] Rietsch J.C., Brender P., Dentzer J., Gadiou R., Vidal L., Vix-Guterl C. (2013). Evidence of water chemisorption during graphite friction under moist conditions. Carbon.

[36] Panitz J., Pope L., Lyons J., Staley D. (1988). The tribological properties of MoS_2_ coatings in vacuum, low relative humidity, and high relative humidity environments. J. Vac. Sci. Technol. A Vac. Surfaces Film.

[37] Zhao X., Perry S.S. (2010). The role of water in modifying friction within MoS_2_ sliding interfaces. ACS Appl. Mater. Interfaces.

[38] Levita G., Restuccia P., Righi M.C. (2016). Graphene and MoS_2_ interacting with water: A comparison by ab initio calculations. Carbon.

[39] Ren X., Yang X., Xie G., He F., Wang R., Zhang C., Guo D., Luo J. (2021). Superlubricity under ultrahigh contact pressure enabled by partially oxidized black phosphorus nanosheets. npj 2D Mater. Appl..

[40] Lü T.Y., Feng H., Zhang Y., Lu Y., Zheng J.C. (2017). Regulate the polarity of phosphorene’s mechanical properties by oxidation. Comput. Mater. Sci..

[41] Reuter K., Scheffler M. (2001). Composition, structure, and stability of *RuO*_2_(110) as a function of oxygen pressure. Phys. Rev. B.

[42] Reuter K., Scheffler M. (2003). Composition and structure of the *RuO*_2_(110) surface in an O_2_ and CO environment: Implications for the catalytic formation of *CO*_2_. Phys. Rev. B.

[43] Stampfl C. (2005). Surface processes and phase transitions from ab initio atomistic thermodynamics and statistical mechanics. Catal. Today.

[44] Ahmad E., Chang H.Y., Kindi M., Joshi G., Cooper K., Lindsay R., Harrison N. (2019). Corrosion Protection through Naturally Occurring Films: New Insights from Iron Carbonate. ACS Appl. Mater. Interfaces.

[45] Soon A., Todorova M., Delley B., Stampfl C. (2007). Thermodynamic stability and structure of copper oxide surfaces: A first-principles investigation. Phys. Rev. B.

[46] Zhao S., Liu X.W., Huo C.F., Li Y.W., Wang J., Jiao H. (2015). Determining surface structure and stability of *ε*-Fe_2_C, *χ*-Fe_5_C_2_, *θ*-Fe_3_C and Fe_4_C phases under carburization environment from combined DFT and atomistic thermodynamic studies. Catal. Struct. React..

[47] Zhao S., Liu X.W., Huo C.F., Li Y.W., Wang J., Jiao H. (2012). Surface morphology of Hägg iron carbide (*χ*-Fe_5_C_2_) from ab initio atomistic thermodynamics. J. Catal..

[48] Zilibotti G., Righi M.C., Ferrario M. (2009). Ab initio study on the surface chemistry and nanotribological properties of passivated diamond surfaces. Phys. Rev. B.

[49] Restuccia P., Ferrario M., Righi M. (2020). Monitoring water and oxygen splitting at graphene edges and folds: Insights into the lubricity of graphitic materials. Carbon.

[50] Wang G., Pandey R., Karna S.P. (2015). Phosphorene oxide: Stability and electronic properties of a novel two-dimensional material. Nanoscale.

[51] Kang S.H., Park J., Woo S., Kwon Y.K. (2019). Two-dimensional dirac fermions on oxidized black phosphorus. Phys. Chem. Chem. Phys..

[52] Kajita S., Righi M.C. (2016). Insigths into the tribochemistry of silicon-doped carbon-based films by ab initio analysis of water–surface interactions. Tribol. Lett..

[53] Levita G., Righi M.C. (2017). Effects of water intercalation and tribochemistry on MoS2 lubricity: An ab initio molecular dynamics investigation. ChemPhysChem.

[54] Giannozzi P., Baroni S., Bonini N., Calandra M., Car R., Cavazzoni C., Ceresoli D., Chiarotti G.L., Cococcioni M., Dabo I. (2009). QUANTUM ESPRESSO: A modular and open-source software project for quantum simulations of materials. J. Phys. Condens. Matter.

[55] Giannozzi P., Andreussi O., Brumme T., Bunau O., Nardelli M.B., Calandra M., Car R., Cavazzoni C., Ceresoli D., Cococcioni M. (2017). Advanced capabilities for materials modelling with Quantum ESPRESSO. J. Physics Condens. Matter.

[56] Giannozzi P., Baseggio O., Bonfà P., Brunato D., Car R., Carnimeo I., Cavazzoni C., De Gironcoli S., Delugas P., Ferrari Ruffino F. (2020). Quantum ESPRESSO toward the exascale. J. Chem. Phys..

[57] Perdew J.P., Burke K., Ernzerhof M. (1996). Generalized gradient approximation made simple. Phys. Rev. Lett..

[58] Grimme S. (2006). Semiempirical GGA-type density functional constructed with a long-range dispersion correction. J. Comput. Chem..

[59] Monkhorst H.J., Pack J.D. (1976). Special points for Brillouin-zone integrations. Phys. Rev. B.

[60] Zilibotti G., Corni S., Righi M.C. (2013). Load-Induced Confinement Activates Diamond Lubrication by Water. Phys. Rev. Lett..

[61] Tang W., Sanville E., Henkelman G. (2009). A grid-based Bader analysis algorithm without lattice bias. J. Phys. Condens. Matter.

[62] Sanville E., Kenny S.D., Smith R., Henkelman G. (2007). Improved grid-based algorithm for Bader charge allocation. J. Comput. Chem..

[63] Yu M., Trinkle D.R. (2011). Accurate and efficient algorithm for Bader charge integration. J. Chem. Phys..

[64] Henkelman G., Arnaldsson A., Jónsson H. (2006). A fast and robust algorithm for Bader decomposition of charge density. Comput. Mater. Sci..

[65] Ta H.T.T., Tran N.V., Tieu A.K., Zhu H., Yu H., Ta T.D. (2021). Computational Tribochemistry: A Review from Classical and Quantum Mechanics Studies. J. Phys. Chem. C.

